# Duality of B Cell-CXCL13 Axis in Tumor Immunology

**DOI:** 10.3389/fimmu.2020.521110

**Published:** 2020-10-21

**Authors:** Angel J. Rubio, Tyrone Porter, Xuemei Zhong

**Affiliations:** ^1^Department of Pharmacology and Experimental Therapeutics, Boston University, Boston, MA, United States; ^2^Department of Biomedical Engineering, University of Texas Austin, Austin, TX, United States; ^3^Hematology and Medical Oncology Section, Department of Medicine, Boston University School of Medicine, Boston, MA, United States

**Keywords:** B cell, CXCL13, tumor immunology, tumor infiltrated immune cells, tumor immune cell interaction

## Abstract

Tumor immunity is a rapidly evolving area of research consisting of many possible permutations of immune cell tumor interactions that are dependent upon cell type, tumor type, and stage in tumor progression. At the same time, the majority of cancer immunotherapies have been focused on modulating the T cell-mediated antitumor immune response and have largely ignored the potential utility that B cells possess with respect to tumor immunity. Therefore, this motivated an exploration into the role that B cells and their accompanying chemokine, CXCL13, play in tumor immunity across multiple tumor types. Both B cells and CXCL13 possess dualistic impacts on tumor progression and tumor immunity which is furthered detail in this review. Specifically, various B cells subtypes are able to suppress or enhance several important immunological functions. Paradoxically, CXCL13 has been shown to drive several pro-growth and invasive signaling pathways across multiple tumor types, while also, correlating with improved survival and immune cell tumor localization in other tumor types. Potential tools for better elucidating the mechanisms by which B cells and CXCL13 impact the antitumor immune response are also discussed. In addition, multiples strategies are proposed for modulating the B cell-CXCL13 axis for cancer immunotherapies.

## Introduction

Recent advances in cancer immunotherapies have highlighted the potential of employing the immune system to impede tumor progression. A major focus has been on employing the T cell-mediated antitumor immune response (1). However, given the complexity of the immune system and known interplay between T cells and B cells, the role of B cells with respect to antitumor immunity should not be overlooked. For instance, B cells have been shown to modulate T cell differentiation and mediate T cell response to tumor antigens (2, 3). Also, B cells possess a diverse array of immunological functions ranging from antibody and cytokine production to phagocytosis of which contribution to tumor immunity is largely unknown (4–8).

Recent studies have explored utilizing antigen presenting cells (APCs) such as dendritic cells (DCs) for cancer immunotherapy (9). B cells also operate as APCs and can be expanded *ex vivo*, so they may too serve as a viable option for cancer immunotherapy (10). Additionally, a significant number of B cells are found in tumors and tumor-draining lymph nodes (TDLNs), localized sites of high immunological activity near tumors (11). However, their exact role remains unclear as contradictory studies have shown that they can be drivers of tumor progression through facilitating immunosuppressive microenvironments or contributors to the antitumor immune response through antigen presentation and T cell activation (10, 11). Therefore, more studies are necessary to elucidate B cells' role in tumor immunity.

Equally important, chemokines dictate proper immune cell trafficking and are being pursued for cancer immunotherapies (12). With respect to tumor immunity, they are becoming increasingly highlighted for their ability to drive immune cell tumor recruitment and impact the tumor-infiltrating lymphocytes (TILs) population. For example, CXCL13 interacts with the chemokine receptor CXCR5 which is present on B cells and some tumor cells and has been implicated as key modulators of both tumor progression and antitumor immunity (13). Studies have shown that CXCL13 can drive tumor growth and invasion through PI3K/AKT signaling or contribute to an enhanced antitumor immune response via increased tumor immune localization (14, 15). Therefore, further inquiry is required to determine its role in tumor immunity. In this review we will delve into the literature of B cells and CXCL13 and attempt to provide the most updated analysis of their roles in tumor immunity. Additionally, we will postulate on their ability to be leveraged for innovative cancer immunotherapies.

## B Cells Subtypes and Functions

The contribution of B cells in tumor immunity remains controversial. However, there is consensus on their importance for generating antibodies which bind to specific antigen epitopes and label them for degradation or targeting (16). B cells function as APCs by utilizing their B cell receptor (BCR) to recognize antigens and are important for presenting foreign and auto-antigens to CD4+ T cells (17–19). B cells can regulate immune system homeostasis through the production of cytokines which modulate T cell differentiation, inflammation, and lymphoid tissue architecture (20).

It is important to define several B cell subtypes to better understand how they may contribute to tumor immunity. For instance, regulatory B cells (Bregs) are important for ensuring proper immunological tolerance and may help combat autoimmunity (21, 22). Bregs can express the immunosuppressive cytokines, IL-10, TGF-β, and IL-35, to impair the activity of DCs and T cells (23). Also, Breg-derived IL-10 is important for promoting regulatory T cells (Tregs), which further contribute to immune suppression (24). Bregs via increased expression of TGF-β1 and IL-10 can induce anergy of CD8+ T cells and apoptosis of CD4+ T cells, respectively (25, 26). With this in mind, it is apparent that Bregs act to dampen immune activity which may contribute to a less robust antitumor immune response. However, because they lack an identifying marker, it is challenging to specifically study their intratumoral functions and interactions with other TILs (27).

Furthermore, conventional recirculating B cells (B2 B cells) can be further stratified into follicular (FO) or marginal zone (MZ) B cells (20). Naive FO B cells reside in lymph node follicles where they present antigens to activated T cells (28). After maturation, they circulate throughout the lymphatic and circulatory system and are the main driver of high-affinity antibody production (29). MZ B cells reside in the spleen MZ where they monitor for blood pathogens utilizing their poly-reactive BCRs (30, 31). They can respond to antigens without assistance from T cells and are able to transport antigens to FO B cells residing in spleen follicles (32).

In contrast, B1 B cells are primarily compartmentalized in the pleural and peritoneal cavities and are hypothesized to be part of innate immune memory (33). They are characterized by their ability to self-renew and constitutively produce natural antibodies (34). These natural antibodies are coded by the germline VDJ sequences and recognize apoptotic cell membranes (35, 36). B1 B cells are also able to phagocytose dying mammalian cells (37). Dysregulation of apoptotic cell clearance can lead to necrosis and inflammation, both of which are associated with tumor progression (38, 39). Therefore, B1 B cells may help mitigate potential inflammatory responses by recognizing and clearing dying cells. Also, B1 B cells can stimulate T cell expansion via CD80/CD86 and promote differentiation of CD4+ T cells (3). Similar to MZ B2 B cells, B1 B cells are capable of eliciting a T cell-independent response (40). B1 B cells differ from B2 B cells, in that they are larger, have resistance to FAS-induced apoptosis, and possess greater *ex vivo* survivability (41). Similar to Bregs, human B1 B cells lack a consensus on their identifying surface markers so studying them in the tumor microenvironment remains difficult (42).

## Evidence of B Cells Promoting Tumor Progression

Given the complex nature of B cells in promoting or suppressing immune response, it is important to detail how they can potentially hamper or promote antitumor immunity. For instance, antibodies can lead to the generation of circulating immune complexes (CIC) which have been associated with poor prognosis in pancreatic ductal adenocarcinoma patients (43). These CIC can suppress the immune response of myeloid cells which then provides an additional barrier to a robust antitumor immune response (44, 45). In prostate cancer, B cell-secreted lymphotoxin (LT) was shown to drive STAT3 signaling to promote tumor growth (46). In a mouse melanoma and lung cancer model, B cells with activated STAT3 contributed to increased tumor growth through the promotion of angiogenesis (47). Additionally, a study showed that B cells can promote bladder cancer metastasis by increasing ECM (extracellular matrix) remodeling gene expression (48).

Furthermore, Bregs TGF-beta production can drive conversion of CD4+ T cells to Tregs leading to inhibition of CD8+ T cells and Natural Killer (NK) cells, both of which are important for limiting tumor growth (49, 50). In a mouse breast cancer model, tumor-evoked Bregs (tBregs) promoted transition of resting CD4+ T cells to Treg cells which correlated with greater metastasis (51). Additionally, tBregs have been shown to elevate myeloid-derived suppressor cells ROS and NO generation leading to CD4+ and CD8+ T cells suppression (52). IL-10 can hamper the production of additional stimulatory cytokines leading to decreased responsiveness of CD8+ T cells, Th1 cells, and NK cells (53, 54). B cells may drive tumor progression through promoting expression of various genes that drive tumorigenesis or by weakening the immune response.

## B Cells' Antitumor Functions and Prognostic Value

In contrast, there is evidence that B cells can be beneficial for enhancing antitumor immunity either directly by interacting with tumor cells or indirectly by assisting additional immune functions. For example, stimulated human B cells *in vitro* have demonstrated the ability to induce lysis of melanoma cells through expression of TRAIL/Apo-2L (55). TIL B cells isolated from breast cancer tissues have been reported to express granzyme B and exhibited *in vitro* cytotoxic activity toward breast cancer cells (56). An additional study has shown that TDLN B cells utilize FasL to directly interact with mammary cancer cells and induce lysis (57). This suggest that B cells may contribute to antitumor immunity by directly killing cancer cells.

In addition, B cells are capable of generating tumor-specific antibodies and have shown to provide protective benefits against breast cancer (16, 58). Additionally, tumor-binding antibodies have been shown to be able to promote tumor cell uptake by DCs (59). Furthermore, in a mouse glioblastoma model, B cell antigen presentation was shown to be essential for T cell-mediated antitumor response (60). The depletion of B cells with anti-CD20 monoclonal antibodies in a melanoma mouse model resulted in hampered CD4+ and CD8+ T cell response (61). Also, activated B cells from cervical cancer patients have been shown to stimulate T cell-mediated antitumor responses (62).

The presence of TIL B cells in multiple cancer types has shown to be a positive prognostic marker for survival. For example, analysis of colorectal cancer tissue samples demonstrated that high B cell infiltration was a good indicator for positive clinical outcome (15). A separate study on human colorectal cancer, determined that TIL B cells were associated with improved patient outcome (63). Likewise, in multiple studies analyzing non-small-cell lung carcinoma (NSCLC) samples, high B cell tumor infiltration generally correlated with better clinical outcomes (64–66). Also, analysis of ovarian tumors showed a positive association between B cell tumor infiltration and patient survival (67).

## CXCL13 Function

CXCL13 is a 10 kilodalton CXC chemokine that is important for mobilizing B cells. Similar to B cells, CXCL13 has been identified as possessing a dualistic impact on tumor progression. For instance, CXCL13 has been associated with metastasis, while at the same time is associated with greater patient survival (15, 68). It is expressed by follicular DCs (FDCs) and helper T cells and is essential for naive B cell homing and organization within lymphoid follicles, sites critical for B cell-antigen interaction and B cell differentiation (69, 70). Also, CXCL13 drives B cell LT expression which in turn promotes increased CXCL13 levels to generate a positive feedback loop (71). CXCL13 through CXCR5 signaling enhances BCR-triggered B-cell activation by altering cell dynamics to enhance antigen gathering at the B cell immune synapse (72). With respect to differences among B cell subtypes, B1 B cells express greater CXCR5 than B2 B cells which may important for ensuring that B1 B cells are recruited and provide localized immunity to the peritoneal cavity (70). This is evidenced in CXCL13-deficient mice which have reduced B1 B cell natural antibody production and response to bacterial antigens in the peritoneum (68). These findings highlight the importance of CXCL13 for regulating B cells.

## CXCL13'S Role in Driving Tumor Progression

CXCL13 has been found to act on cancer cells and potentially drive tumor progression. The addition of CXCL13 to breast cancer cells *in vitro* increased expression of matrix metalloproteinase-9 (MMP-9) and genes reasonable for driving the epithelial to mesenchymal transition (EMT) (73). MMP-9 is important for ECM remodeling and invasion of tumor cells through the basement membrane. Additionally, the EMT is a common step in tumor progression for epithelial cell cancers (74). CXCL13 administration to oral squamous cell carcinomas (OSCC) resulted in heightened CXCR5 and MMP-9 expression (75). Similarly, in prostate cancer cell lines, the addition of CXCL13 increased expression of ECM remodeling genes (76). Also, CXCL13 through CXCR5 was shown to promote growth and invasion via the PI3K/AKT pathway in clear cell renal carcinoma (14). In liver cancer, CXCL13 has been shown to activate the pro-growth Wnt/B-catenin signaling pathway (77). Furthermore, CXCL13 may have a specific role in promoting bone metastasis. CXCL13 knockdown resulted in reduced prostate cancer and OSCC bone invasion in mouse models (75, 76).

With respect to hematological cancers, specifically, B cell chronic lymphocytic leukemia (B-CLL) and acute lymphocytic leukemia (B-ALL), there is significant evidence that CXCL13 drives pro-growth and survival signaling (78, 79). Additionally, increased serum CXCL13 levels were found to be associated with greater risk of B cell non-Hodgkin's lymphoma (NHL) in HIV-infected individuals (80). Given CXCL13's impact on B cells, it is somewhat intuitive that it would exacerbate B cell malignancies. In summary, tumor cells may utilize CXCL13 to promote growth, invasion, and metastasis.

## CXCL13'S Role in Enhancing Antitumor Immunity

In contrast, CXCL13 may contribute to a greater antitumor immune response through improving immune cell tumor infiltration. For instance, in human breast cancer tumor tissues greater CXCL13 expression was linked with increased T cell and B cell tumor recruitment (81). Furthermore, among colorectal cancer patients, increased intratumoral CXCL13 correlated with greater T cell and B cell tumor infiltration and prolonged patient survival (15). CXCL13 administration into the colonic submucosa of mice with colorectal cancer resulted in decreased tumor growth (15). Additionally, in patients with HER2+ breast cancer increased CXCL13 correlated with better survival (82). High expression of CXCL13 among triple negative breast cancer (TNBC) patients corresponded with better outcomes (83). Likewise, analysis of genome-wide cDNA expression of tumor samples from ovarian cancer patients revealed that high CXCL13 correlated with better prognosis (84). CXCL13 may hinder tumor progression by increasing the number of immune cells at the tumor site which is evidenced by its correlation with greater prognosis and survival in multiple tumor types. The complex impact of CXCL13 on tumor immunity is summarized in Table 1.

**Table 1:** CXCL13 impact across different types of cancer.

**Cancer type**	**Pro-tumor**	**Antitumor**
Breast cancer	*In vitro* addition to MDA-MB-231 cells increases MMP expression Model: Mouse cancer cells (68)	Correlated with greater survival and immune tumor infiltration Model: *ex vivo* analysis of human breast cancer tissue (81) HER2+/TNBC: Correlated with better prognosis Model: Human clinical data (82, 83)
Genitourinary cancers	Clear cell renal carcinoma: Promotes PI3K/AKT signaling (CXCR5+) Model: human tissue analysis (14) Prostate cancer: Enhances ECM remodeling and bone metastasis (CXCR5+) Model: human engineered cell lines and xenograft mouse model (76)	Ovarian cancer: Correlated with better prognosis Model: Human healthy and cancerous tissue cDNA expression data (84)
Colorectal cancer	N/A	Correlated with improved survival Model: Human clinical data (15)
OSCC	Increases MMP expression and bone invasion (CXCR5+) Model: Human-derived OSCCs cells and xenograft mouse model (75)	N/A
Liver cancer	Liver Cancer: Drives Wnt signaling (CXCR5+) Model: *in vitro* analysis of human samples (77)	N/A
Leukemia	B-CLL and B-ALL: Promotes apoptosis resistance Model: *in vitro* analysis of human samples (78, 79)	N/A

## Potential of B Cells and CXCL13 in Cancer Immunotherapy

B cells as well as CXCL13 play multifunctional roles in tumor immunity. Careful modulation of each may prove to be effective for bolstering existing cancer immunotherapies or for designing novel stand-alone treatments. For example, B cells have multiple characteristics such as ability to be readily expanded *ex vivo*, produce antibodies and present antigens to T cells which make them a viable option for adoptive cell transfer therapy (10). B cells isolated from TDLNs of mice inoculated with breast cancer cells were activated *ex vivo* and administered to mice with breast cancer (85). The activated B cells were able to induce tumor-specific T cell immunity and prevent lung metastases. Also, the activated B cells in combination with activated T cells resulted in tumor regression demonstrating the therapeutic potential of using B cells. An additional study showed that stimulated B cells could be employed for cross-presenting tumor-specific antigens to T cells (86). In this study, activated B cells loaded with tumor antigen were capable of impeding tumor growth, demonstrating that B cells can potentially be a stand-alone treatment option. Alternatively, inhibiting Breg cell activity may be beneficial for targeting tumors with immunosuppressive microenvironments. This would be most useful in tumors with high Breg infiltration.

Additionally, CXCL13 may be utilized to improve the antitumor immune response by increasing B cell localization to tumor site. In humans, CXCL13 has been shown to increase B cell tumor infiltration and correlate with prolonged survival in multiple tumor types (82–84). In mouse colorectal cancer studies, direct CXCL13 administration was effective for impeding tumor growth (15). Also, CXCL13 can initiate a positive feedback loop for B cell activation so it may useful to deliver CXCL13 into the tumor to enhance TIL B cell antitumor functions (71). Additionally, CXCL13 can be utilized to selectively target B cells. CpG-oligodeoxynucleotides (ODNs) conjugated to CXCL13 for B cell-specific delivery resulted in enhanced B cells activation of CD8+ T cells and reduced lung metastasis (87). CXCL13 can be similarly employed to deliver inhibitory agents to Bregs to combat intratumoral immune suppression. Also, CXCL13 can potentially be used in conjunction with B cell-based immunotherapies to improve B cell tumor localization and result in greater efficacy. In contrast, for CXCR5+ tumors, antibody blockade of CXCL13 may be a useful strategy for preventing CXCL13-driven tumor growth and invasion. For instance, administration of anti-CXCL13 to MDA-MB-231 cells *in vitro* resulted in apoptosis (88).

## Discussion

Although B cells' role in tumor immunity is quite complex, it is apparent that they are important modulators as they are capable of both hindering or promoting antitumor immunity. Specifically, Breg activity may drive an immunosuppressive tumor microenvironment by suppressing T cell and DC activity (21, 23). In contrast, B1 B cells may contribute to a more effective antitumor immune response by enhancing T cell-mediated immune response (3). However, for both B cell subtypes there is a lack a consensus on their human surface markers and, therefore, they are difficult to study within the context of cancer (21, 42).

Additional tools are needed to accurately analyze B cell subtypes within tumor microenvironments. In addition to surface markers, functional *ex vivo* assays on isolated TIL-B cell subtypes may help further define roles. For instance, isolated TIL-B cells could be co-cultured with CD4+ and CD8+ T cells and monitored using the modulation of T cells as a readout. If specific TIL-B cells are identified as stimulating T cell activity, then they can be furthered genotyped to identify gene expression patterns that reflect the change in cell status and functions during the immune response. Similarly, CXCL13's role in tumor immunity is contradictory (82, 88). Analyzing the amount of intratumoral CXCL13 after tumors have been excised does not clearly decipher if CXCL13 is a driver or responder to tumor progression. Instead, new tools that enable real-time tracking of CXCL13 expression during tumor progression are necessary to more accurately address this inquiry. Ultimately, B cells and CXCL13 have great therapeutic potential for cancer treatments. However, researchers must be cautious as both must be considered with respect to tumor type and CXCR5 tumor expression. It is pertinent that information on the B cell tumor population and tumor-immune microenvironment is known before pursuing this type of immunotherapy.

## Author Contributions

AR wrote the initial draft of the manuscript. XZ and TP contributed to the editing and revising of this work. All authors contributed to the article and approved the submitted version.

## Conflict of Interest

The authors declare that the research was conducted in the absence of any commercial or financial relationships that could be construed as a potential conflict of interest.
